# Perspectives on adherence to prescribed home exercises after polytrauma: A qualitative study

**DOI:** 10.4102/sajp.v81i1.2163

**Published:** 2025-06-11

**Authors:** Keamogetswe Monaiwa, Mariatha Yazbek, Nontembiso Magida

**Affiliations:** 1 Department of Physiotherapy, Faculty of Healthcare Sciences, University of Pretoria, Pretoria, South Africa; 2 Department of Nursing Science, Faculty of Healthcare Science, University of Pretoria, Pretoria, South Africa

**Keywords:** international classification of functioning and disability, adherence, polytrauma, home exercise programme, physiotherapy, facilitators, barriers

## Abstract

**Background:**

Polytrauma can be life altering, requiring a holistic approach to reach the highest functional level. Physiotherapists prescribe home exercise programmes (HEPs) to prevent complications associated with immobility. Adherence to HEPs is crucial, yet factors influencing non-adherence remain underexplored.

**Objectives:**

Our study explored patients with polytrauma perspectives on adherence to prescribed HEPs.

**Method:**

A qualitative exploratory, descriptive design was used to recruit participants purposively from a rehabilitation unit in Tshwane district, South Africa. Participants diagnosed with polytrauma, aged 18 years or older, and prescribed a HEP were included, while those with cognitive impairments or language barriers were excluded. Consent was obtained while hospitalised, and participants were contacted 3 months post-discharge for telephonic semistructured interviews lasting 30–45 min. Ethical clearance (reference number: 595/2022) and institutional permission were granted. Interviews were audio-recorded and conducted until data were saturated. Data were verbatim transcribed and analysed thematically to identify key themes and sub-themes.

**Results:**

Thirteen participants (8 male and 5 female participants) with a mean age of 43.77 (standard deviation = 10.45) were interviewed. The four major themes were physical, psycho-cognitive, social and environmental factors. Adherence barriers were more significant than facilitators. The most frequently reported facilitator was family support, whereas the most commonly reported barrier was pain.

**Conclusion:**

Polytrauma patients identified more barriers than facilitators affecting HEP adherence. Pain significantly hindered adherence, while family support was a key enabler.

**Clinical implication:**

Physiotherapists should work collaboratively with patients to develop inclusive HEPs that consider their demographic, social, psychological, physical and environmental context.

## Introduction

Polytrauma is defined as injuries that have occurred in more than one bodily system where there is the presence of traumatic shock or haemorrhage with the potential to endanger vital body functions (Butcher & Balogh 2014). A trauma experience may be a blunt or penetrating injury piercing the body and may lead to multiple injuries on different bodily systems (Dattatri et al. 2021). The international consensus concludes that anatomical and physiological parameters should be considered when defining polytrauma (Butcher & Balogh 2014). A polytrauma experience can be life changing, as injuries can range from moderate to severe, requiring intensive medical, surgical and therapeutic intervention. Trauma is the leading cause of mortality worldwide (Parker et al. 2021). The burden of polytrauma remains high in the sub-Saharan African region, with an estimated mortality rate of 4.2% (Tyson et al. 2015). Over 90% of trauma-related deaths reported occur in low- to middle-income countries, and Africa has the highest trauma-associated mortality and disability rate (Collins et al. 2022). To reduce disability and improve quality of life, physical rehabilitation is shown to have benefits for those diagnosed with polytrauma (Zwingmann et al. 2016). Physical rehabilitation also helps re-integrate individuals into their home, community and workplace environments (Simmel 2018).

The overall goal for physical rehabilitation is centred around the International Classification of Functioning and Disability (ICF), which is based on integrating the medical and social models of disability (Armstrong, Champagne & Mortimer 2019). Furthermore, the ICF conceptualises one’s level of functioning with their impairments and environmental and personal factors. Having an expert multidisciplinary team (MDT) within a physical rehabilitation facility prioritising the ICF model has improved clinical outcomes in patients with polytrauma (Dattatri et al. 2021). Physiotherapists within the MDT prescribe home exercise programmes (HEPs) for patients before their discharge from the rehabilitation unit (Högstedt 2023). Home exercise programmes help ensure the continuation of therapy within a patient’s home environment. Adherence to a prescribed HEP has been shown to improve clinical outcomes and decrease the overall burden of care upon caregivers (Suikkanen et al. 2021). Patients who fail to adhere to a prescribed HEP may prolong the duration of their disease management by directly impacting their functioning level and decreasing their physical independence (L’heureux et al. 2020). Studies that explore perceptions of patients with polytrauma regarding their prescribed home exercise adherence were not found; hence, our study aimed to explore the perspectives of patients with polytrauma regarding adherence to their prescribed HEP.

## Research methods and design

### Study design

Our study adhered to the standard for reporting qualitative research (SRQR) guidelines (Sinha et al. 2024b). Our study employed an exploratory qualitative descriptive research design to describe subjective perspectives (Kim et al. 2020). The author determined that an exploratory design is best suited to address the research aim as it may help produce responses that offer human experiences (Kim et al. 2020). Exploratory design will also expand people’s knowledge and comprehension of society and create the everyday realities of social phenomena (Dattatri et al. 2021).

### Study setting

Our study was conducted at a selected private hospital rehabilitation unit at the heart of Tshwane district in Gauteng Province, South Africa. The private hospital has 386 beds, while the rehabilitation unit has 21 beds and a gym area. The unit admits patients with varying diagnoses and injuries, referred from surrounding hospitals. The average polytrauma admission rate at the rehabilitation unit is approximately 30% of total admissions. The rehabilitation unit consists of an MDT, which includes physiotherapists, occupational therapists, rehabilitation nurses, doctors, speech therapists, social workers, psychologists, dieticians and a rehabilitation admissions consultant. The patients admitted to the unit are of all races, and the primary payment source is medical aid.

### Participant sampling and recruitment strategy

Following ethical clearance approval from the University of Pretoria, the author sought ethical approval from the private hospital to conduct our study within the rehabilitation unit. Once permission was granted, the participants were screened and selected based on their diagnosis, and consent was sought from participants who met the inclusion criteria while still in the rehabilitation unit. Participants were purposively sampled (Campbell et al. 2020) and recruited at different time frames of their respective rehabilitation programmes, depending on their date of admission and discharge. The purposive sampling technique involved non-probability sampling of a homogeneous group, with the intention to select participants diagnosed with polytrauma, and those with a prescribed HEP by a physiotherapist. We included participants who were diagnosed with polytrauma and were 18 years or older. Participants selected for our study had gone through the rehabilitation programme. We excluded participants who were diagnosed with traumatic brain injury and altered speech as they may have changed cognitive function or poor communication, which may impact their ability to participate in our study.

Participants were enrolled prior discharge to ensure that the research adhered to the Protection of *Personal Information (POPI) Act*. Once written consent was given, participants’ personal and contact details were captured. Participants were informed about the nature of our study as well as its aim. However, the objectives of our study were not disclosed to the participants to prevent any bias on the results of our study. Three months after discharge, the patients were contacted to arrange a semistructured telephone interview at their convenience. Telephonic interviews were held with study participants. Telephonic interviews were deemed fit for our study as participants were from different provinces, with one participant from Germany. Some participants were prohibited from driving because of the nature of their injuries. As a result, face-to-face interviews would have posed a logistical challenge. Permission to audio-record the telephone interview was requested and granted by the participants. During the pilot study, an experienced qualitative researcher, N.M. trained K.M. to conduct semistructured interviews, and K.M. conducted all of the interviews. Reflective notes were taken in a diary during the data collection process. Our study was conducted using a social constructivist methodology. This claims that reality is subjective to each individual and socially constructed (Attia & Edge 2017). Consequently, the reflective diary was used to detect and acknowledge any possible interpretation bias and to help provide congruence across the data. Although some participants continued with outpatient physiotherapy in their respective capacity, the rehabilitation professionals who were involved in their care during in-patient rehabilitation did not provide further outpatient therapy or any rehabilitative service or care.

### Data collection instrument and procedure

A 3-month post-discharge period was given to the participants to allow them to perform the individualised HEP prescribed by their treating physiotherapists. Semistructured interview questions were generated from our study objectives and literature (Dejonckheere & Vaughn 2019). A peer review panel from the university conducted internal testing of the preliminary interview guide to check for ambiguous or unnecessary questions. The first two patients discharged from the rehabilitation unit were piloted telephonically using the semistructured interview guide to ensure all objectives were covered and if there was a need for reformulating questions (Malmqvist et al. 2019). The duration of the pilot study interviews was 33 and 37 min, respectively. The interviews were conducted in participants’ home language (IsiZulu, Setswana and English). A few terms were rephrased following the analysis of the data from the pilot study to enhance its quality. The results of the pilot study were not used in the main study.

Subsequently, the author contacted the participants telephonically 3 months post-discharge, and semistructured telephone interviews were conducted. The participants were informed that the interview would be audio-recorded on their consent forms before the interviews. During each interview, K.M. asked open-ended questions guided by the interview framework, which was developed in accordance with the ICF framework. This approach enabled participants to share their perspectives while aligning with the principles of the ICF framework. The author probed the interviewee to elaborate on the original response or to follow a line of inquiry if the responses were short and unclear (Dejonckheere & Vaughn 2019). The duration of the interviews varied from 30 to 45 min long. While some could share much information regarding their HEPs, others were not as expressive. It is important to note that some participants of our study received HEP prescribed by other multi-disciplinary members, according to their rehabilitation needs.

### Data processing and analysis

Data were verbatim transcribed by K.M. and confirmed by N.M., who verified the transcripts by rereading and listening to the audio recording. The transcripts were sent back to the participants via phone for verification so that additional member checking could be conducted. All data were made anonymous by eliminating names from the transcripts. Audio recordings and identifying information were stored on a password-protected computer at the university’s Department of Physiotherapy to avoid a confidentiality breach. Altas.ti version 23 software was used to organize data and facilitate the coding process. Atlas.ti was developed by Thomas Muhr, based in Berlin, Germany (Ronzani et al. 2020). Deductive thematic analysis was used to analyse interview data to understand participants’ diverse perspectives (Dawadi 2020).

Using the Atlas.ti software, themes and sub-themes were produced, and the ICF framework’s guidance was followed to refine the sub-themes through deductive (concept-driven) coding (Dawadi 2020). The research process involved identifying, analysing and reporting patterns (themes) within the data to capture data that answers our study objectives. All authors discussed, compared and consolidated the codes into themes.

### Trustworthiness

To ensure credibility, the interview transcripts were reviewed by the transcriber and then read and reread by another researcher (Shufutinsky 2020). Transferability was facilitated by thoroughly explaining participants, contexts and research methodologies employed in this investigation. Dependability was secured by including standout quotes from most participants to bolster the emerging themes (Jafree & Barlow 2023). Confirmability was ensured by providing the participants with the transcripts to verify the data’s accuracy (Sinha et al. 2024a).

### Ethical considerations

Ethical clearance and permission to conduct our study were sought from the University of Pretoria Research and Ethics Committee (reference number: 595/2022). Written permission was sought from the designated private hospital where the rehabilitation unit is located. Our study followed the principles of the Helsinki Declaration as outlined by Kurihara et al. (2024).

## Results

### Demographic profile of the individuals

Demographic information of our study participants is presented in Table 1. Thirteen participants participated in our study, as seen in Table 1. There were more male (*n* = 8) than female (*n* = 5) participants. The age group varied between 31 years and 59 years, with a mean age of 43.77 (standard deviation = 10.45). Two participants were retired, one was unemployed and 11 were employed at the time of data collection. The most commonly reported injuries among participants included femur fractures, tibia and fibula fractures and spinal fractures, with two participants having sustained a spinal cord injury.

**TABLE 1:** Demographic profile of participants (*N* = 13).

Participant code	Gender	Age (years)	Employment status	Injuries sustained
P01	Female	37	Employed	Distal femur fracture, proximal tibia and ankle fracture.
P02	Male	59	Employed	Clavicle and rib fractures, left haemothorax, pulmonary contusions, left radioulnar fracture, left acetabulum, right SI joint diastasis bilateral pubic ramus, left eye injury, liver and spleen injury and L1–L5 spinal fracture.
P03	Male	54	Employed	L1–L5 spinal fracture, pelvic fracture and multiple rib fractures.
P04	Male	34	Employed	Proximal tibiofibular fracture, lung contusions and rib fractures.
P05	Male	48	Employed	Proximal femur fracture, L4–S1 spinal fracture and rib fracture.
P06	Female	48	Retired	Proximal tibia fracture, pelvic fracture, patella fracture, multifocal fibula fracture.
P07	Female	48	Retired	Left clavicle fracture, left scapula fracture, rib fracture, right radius fracture, 3–5 metacarpal fracture, right femur fracture.
P08	Female	58	Employed	Left clavicle fracture, left acetabular fracture, left radioulnar fracture.
P09	Male	31	Employed	Left patellar fracture and left proximal tibia fracture.
P10	Male	27	Employed	Left clavicle fracture, right distal femur fracture, patella fracture, right distal tibia fracture and multiple rib fractures.
P11	Female	35	Unemployed	Right metacarpophalangeal fracture, proximal radioulnar fracture and distal femur fracture.
P12	Male	54	Employed	Left tibiofibular fractures, multiple rib fractures and right hip dislocation fractures.
P13	Male	36	Employed	T12–L1 spinal fracture, multiple rib fractures and proximal humerus fracture.

Table 2 represents four themes and their respective sub-themes that were identified from our study. Themes related to bodily functions and structure, as well as environmental and participation factors, were identified. Some aspects of the theme of psycho-cognitive factors relate to the category of personal factors within the ICF framework. The robustness of the analysis was assessed using Braun and Clarke (2006) 15-item checklist.

**TABLE 2:** Summary of themes, sub-themes and verbatim quotes.

Themes	Sub-themes	Verbatim quotes
Physical factors	Musculoskeletal pain	‘You know, with some exercises, you would find that I would feel a little pain and then leave them halfway, but when I am with the physio, they can tell me what to do and what not to do so that I do not feel much pain.’ P02 ‘I have pain in my leg. My leg feels very sore. I cannot do the exercises.’ P08 ‘But this hip replacement is painful, even if I exercise. I’m getting so much pain.’ P11
	Range of motion	‘Yes, I do some of the exercises, but not the others, because the knee does not become easily flexible, so it is a bit hard.’ P02 ‘I try to do squats even though I can’t bend the leg too much …’ P08 ‘Yes, I remember, but my right hip still can’t move fully up and to the side …’ P10
	Muscle weakness	‘The exercise I got for my left leg is challenging because my muscles are very weak, I can’t control it, and it’s hard for me to do the exercise.’ P08 ‘… because I could not lift this side well [*referring to left arm*], I would try, but I could not do it to the level I am supposed to.’ P12
Psycho-cognitive variables	Memory	‘No, truly speaking, I do not do my exercises because I do not remember them. I don’t know why I could not remember my exercises.’ P01 ‘But to be honest, I cannot remember some exercises. I don’t know if it is because they were too hard for me to do, but I can’t remember them.’ P08
	Fatigue and exhaustion	‘I was getting tired sometimes. Easily getting tired, so I only managed to do it twice a day.’ P11 ‘Honestly, I don’t do exercises because sometimes it’s just me feeling lazy.’ P04 ‘Let me not make the pains an excuse; sometimes I would just be lazy.’ P13
	Facets of motivation	‘I would say that I had a lack of motivation due to most of the focus being placed on my sickly husband. They attended to him more than me at the time.’ P07 ‘But once I sit alone, not talking to anyone, there is nothing positive that I think about; I become demotivated to exercise and get better.’ P12 ‘The children at home motivate me; sometimes I realise that sitting like this, the more I don’t exercise, I will have contractures and what-not, and those kinds of things will delay me and make me worse.’ P1
	Stress and low mood	‘Those I was doing at home were inconsistent because of the situation with my sick husband here. It was stressful seeing him sick.’ P07 ‘I would want something, and then it doesn’t happen the way I want it, then I start getting stressed and decide to sleep all day because right now I live in pain.’ P13 ‘When my family helps me exercise, I feel small and burdensome. And that makes me feel down.’ P11
Environmental factors	Weather conditions	‘The one of walking, I was walking, but what would disturb me is the weather; you would find that it’s too cold, I cannot walk in the street, and I could not do it as I am supposed to.’ P12 ‘Sometimes, it is difficult to do leg exercises when cold. Because the cold causes the leg to be in pain.’ P03
	Home environment	‘Sometimes, you find that there isn’t enough space in the house. Our house is tiny, so doing those exercises can be difficult.’ P07 ‘My home environment and the people motivate me to exercise. I have what I need at home to do my exercises.’ P12
Social factors	Family support and burden	‘When the schools were closed, they helped me, but now that I am alone, there is no one to assist me.’ P02 ‘Sometimes I find the exercises hard, and I need assistance. But I live alone.’ P10 ‘My partner helps me to do it right. He encourages me to have a positive attitude towards my exercises.’ P11 ‘I need help with my exercises. I feel like a burden when my children are always helping me with my exercises.’ P08
	Work and time constraints	‘My challenge is, you see, now my shifts at work are 12 hours, including night shifts. So, I do my exercise but not as frequently.’ P04 ‘Those times I used to do them every day, but now that I am back at work.’ P05 ‘During the week, my work is demanding, and I cannot do my exercises. I cannot find time to exercise on weekends because of responsibilities at home.’ P02

Four themes emerged from our study, and 10 sub-themes further provided a breakdown of the participants’ perspectives regarding their HEP. Themes were categorised into physical, psycho-cognitive, environmental and social factors.

### Barriers to adherence to prescribed home exercise programme

#### Physical factors

Physical factors are bodily experiences that either promote or hinder the quality of movement, function and performance in daily activities. Factors include symptoms that involve dysfunction of the muscles, joints and connective tissue. Bodily experiences can promote or inhibit exercise participation and overall quality of life. For these participants, pain, limited range of motion and muscle weakness decreased adherence to their prescribed HEP. Pain was one of the most common barriers to adherence to exercise, as described by our study participants. While some participants managed to do some exercises, they could not complete the entire exercise regime. One of the participants referred to pain felt in the lower limb and the muscle, alluding to her inability to do her HEP. Although pain and tiredness were vaguely described as disabling, they resulted in decreased adherence to the prescribed HEP. Some reported difficulties in performing their HEP because of limitations of previous joint operations.

Some participants expressed a decreased range of motion (ROM) of the knee and hip joints, compromising the quality of the prescribed exercises. Body stiffness was also reported, which resulted in low motivation to perform exercises. Although the stiffness may or may not have been directed to a specific joint or bodily area, it resulted in decreased mobility, with an overall effect of poor adherence. Another physical contributor to poor exercise adherence was muscle weakness, reported as an inability to stand up from the floor. In contrast, others complained about the difficulty of lifting their upper or lower limbs, which reduced their ability to perform the prescribed exercises.

#### Psycho-cognitive variables

Participants who had psycho-cognitive complaints expressed limitations in their memory and feelings of fatigue and exhaustion. Exercise adherence is primarily influenced by the ability or inability to accurately demonstrate and recall the prescribed HEP (Peek et al. 2019). Confusion in the prescribed HEP was also stated as a barrier. Consequently, these exercises were excluded from their regimen. This experience highlights the potential impact of exercise complexity on adherence to prescribed HEPs. Poor adherence to their HEPs was a result of some participants reporting feelings of exhaustion, which were typified by apathy and fatigue. As motivation affects a person’s capacity for learning, memory and task performance, it is a crucial component of psycho-cognitive functioning. According to the participants, one major obstacle to following their recommended exercise regimens is a lack of motivation. Additionally, it was noted that stress, family issues and poor mood all contributed to decreased time spent exercising at home.

#### Social factors

The lack of family support significantly influenced poor adherence to recommended exercises. Some participants expressed a need for physical assistance from family members to perform specific exercises effectively, while others required verbal encouragement and support from those they lived with. Conversely, some participants claimed to have received too much physical support, which caused them to feel like a burden to their families. This belief frequently deterred them from getting assistance, even when required. Exercise adherence was also found to be hampered by personal and professional obligations. These obligations often interfered with developing new routines and adopting lifestyle modifications, such as regular exercise. Although returning to work after an injury is good, some participants found that their work schedules did not align with the HEPs they were prescribed, making it difficult to follow. The demands of their jobs prevented them from regularly participating in exercises, even though the nature of their employment was not made clear. Likewise, domestic duties like helping kids with their homework and taking care of chores further limited the amount of time that could be spent exercising. Poor adherence to the recommended HEP resulted from these obligations added together.

#### Environmental factors

The home environment was described to play a significant role in exercise adherence for some study participants. Participants referred to having limited access to resources and equipment in their homes to facilitate the execution of prescribed exercises. Equipment included not having the correct chair to perform some exercises in a sitting position or the chair being too low to stand up from. Some participants reported having disadvantageous surroundings, such as insufficient space in the house, which prevented them from doing some exercises. Another aspect of environmental factors included extreme weather conditions and changes. Some participants complained about an inability to exercise during humid conditions, while others found exercise problematic during cold temperatures. The heat made it difficult for some participants to actively engage in their exercises, especially those requiring an outdoor environment. With chilly temperatures, some participants complained about painful joints when performing exercises that involved large joints such as the hips and knees. As a result, such weather changes resulted in poor exercise adherence.

#### Facilitators to adhere to prescribed home exercise programme

In our study, participants reported fewer exercise adherence facilitators. One participant cited high self-efficacy and discipline as the main reasons why they completed their HEP. Another participant mentioned the possibility of returning to work as a key motivator. In contrast, several others emphasised the desire to regain premorbid functioning as a significant source of motivation. Social support was found to be a key facilitator in supporting HEP adherence. Increased adherence was also found among participants whose family members actively participated in their exercise programme. Family members’ verbal support and affirmation promoted a positive attitude towards the HEP, which improved compliance even more. Factors that improved adherence were found to be minimally reported by the participants. Furthermore, facilitators of HEP adherence were found to be externally influenced rather than internally determined.

## Discussion

Our study aimed to explore the perspective of patients with polytrauma regarding adherence to HEP. Participants in our study ranged widely in terms of gender and age. The sample ranged in age from early adulthood to late middle age, with a greater proportion of men than women. This distribution sheds light on our study population’s demographics, which could affect the results and how well they apply to comparable groups. Internationally, female participants aged between 16 years and 44 years are associated with a lower mortality rate following polytrauma injuries (Pape et al. 2019). A study in Ghana found that polytrauma was marked higher in younger than in older people (Seidu, Alhassan & Buunaaim 2024). Furthermore, rehabilitation for people with polytrauma injuries remains sub-optimal nationally (El-Tallawy et al. 2021).

Adherence to a prescribed HEP among patients diagnosed with polytrauma is multi-faceted and influenced by various factors, which may pose as barriers or facilitators. In our study, more barriers were reported than facilitators. Common barriers brought forth by participants were diverse and categorised into physical, psycho-cognitive, environmental and social factors. Facilitators existed within the categories of psycho-cognitive, social and environmental factors. Musculoskeletal pain is pain or significant discomfort arising from musculoskeletal structures such as muscles, bones, joints, ligaments and tendons (Khoja et al. 2022). The pain experience is persistent and often debilitating and has the likelihood of negatively impacting participation in activities of daily living. Although it can be acute, the pain and discomfort described by these participants were mainly chronic. Musculoskeletal pain is prevalent among patients with polytrauma and can develop into chronic pain syndromes that can be difficult to treat and manage (El-Tallawy et al. 2021). Study participants in several body parts reported musculoskeletal pain. Pain experience was persistent and debilitating and often interfering with activities of daily living. According to Bakaa et al. (2021), musculoskeletal pain was found to be a barrier among patients with total knee arthroplasty. A similar study in England reported low adherence to prescribed HEP among persons with persistent musculoskeletal pain (Meade, Bearne & Godfrey 2021). Holt et al. (2020) add that the experience of pain during exercise acted as a barrier to home exercise therapy among youth and adults with musculoskeletal conditions.

Our findings reported decreased ROM and bodily stiffness as barriers to HEP adherence. A decrease in ROM was experienced in the hip and knee joints, resulting in great difficulty when executing some prescribed exercises. Range of motion refers to the degree or extent to which a joint can move through its standard path(s) (Sanz et al. 2020). A decrease in ROM occurs when the movement of a joint is restricted, resulting in limited flexibility and mobility. Polytrauma often leads to prolonged hospital and rehabilitation stays. Oftentimes, injuries sustained may require limited mobility, prolonged bed rest and weight-bearing restrictions. Chen, Hu and Tan (2020) supported the findings, stating that limited ROM is a barrier when exercising regularly, affecting persons of various age groups and fitness levels. Literature suggests that exercise therapy improves ROM among stroke patients and has the potential to manage chronic pain experienced (Srinayanti et al. 2021).

Our study reported muscle weakness as a barrier to exercise adherence. Muscle weakness, also known as muscle hypotonia, is a physiological state characterised by decreased strength and power, affecting the neuromuscular junction (Holt et al. 2020). A study in the United States of America (USA) found that muscle weakness among patients with lower limb osteoarthritis led to poor exercise adherence (Cinthuja, Krishnamoorthy & Shivapatham 2022). A similar study reported that muscle weakness and muscle atrophy are major contributors to decreased exercise tolerance and adherence among patients with knee osteoarthritis (Ledingham et al. 2020). Although participants who complained of muscle weakness in our study did not suffer from osteoarthritis, there is a link between the presence of muscle weakness and poor exercise adherence. According to Sanz et al. (2020), polytrauma patients experience muscle weakness during the presence of musculoskeletal pain, resulting in decreased mobility and function.

Psycho-cognitive barriers existed within the population of our study. Psycho-cognitive factors included the inability to remember exercises, fatigue and exhaustion, facets of motivation, stress and low mood. The ability or inability of one to remember prescribed exercises ultimately affects whether one is likely to adhere to the programme. Participants’ ability to complete the exercises depended on several variables, including whether the programme was provided verbally or on paper. All participants in our study received printed HEPs. Consequently, their inability to recall their exercises suggested that some may have misplaced or lost their HEP instruction sheet. Ultimately, these patients reported poor adherence to their HEPs. Poor memory and forgetfulness were found to be barriers to exercise adherence among patients with various diagnoses (Mahmood et al. 2023). A study by Zhen et al. (2020) also reports that forgetfulness is a barrier to successful adherence to a HEP.

These participants described fatigue and exhaustion as tiredness, laziness and low energy, which may be associated with the extra effort and attention required to do simple activities. While these terms can refer to physical and mental well-being, participants in our study primarily addressed mental well-being. Furthermore, the feelings of fatigue and exhaustion were barriers to HEP adherence. In a study that evaluated exercise adherence among breast cancer survivors, exhaustion was a significant barrier to exercise participation (Kim et al. 2020). Although the term ‘laziness’ can broadly describe how one truly feels, Lang (2024) reported that laziness significantly contributed to poor exercise adherence among patients diagnosed with musculoskeletal disorders.

Psychological and cognitive variables influence an individual’s mental processing, behaviour and overall well-being (Van Agteren et al. 2021). According to the ICF framework, personal factors may encourage or restrict participation among persons with disability (Ritschel et al. 2021). Personal factors are regarded as individualised and specific to an individual, potentially altering one’s mental well-being and cognitive functioning. Participants cited stress and low mood as the leading causes of their failure to follow their HEP. These elements significantly impact physical, mental and emotional health. According to Darroch et al. (2020), post-traumatic stress disorder is frequently present among polytrauma patients, and it negatively affects adherence to regimens given. Furthermore, it is typical for these patients to experience emotional setbacks upon discharge as they attempt to re-integrate into their homes and communities (Chadwick & Billings 2022). Fatigue and exhaustion affect physical and mental well-being (Behrens et al. 2023).

The social circumstances of some study participants impacted their ability to adhere to their exercise programme. The absence or lack of family support resulted in poor exercise adherence and, ultimately, the lack of desire to reach complete functional independence within their home environment. Physical activity is shaped by multiple levels of influence within one’s social environment (Roberts et al. 2024). Adherence to home-based exercises requires the active involvement of family members within the home and a supportive social network (Shelton et al. 2011). Ekegren et al. (2020) report that the absence of family and peer support among patients with post-traumatic diagnoses became a barrier to home exercises. Similarly, a study in Switzerland found that poor social support from family members or friends was one of the significant barriers to adherence to HEPs prescribed among patients with varying diagnoses (Bachmann, Oesch & Bachmann 2018).

The participants described a lack of motivation as one of the leading factors that led to poor adherence to their prescribed exercise programme. The main factors that led to low motivation were living independently at home and the absence of a spouse or a family member. Another qualitative study demonstrated that high self-motivation and personal discipline facilitated exercise adherence among trauma patients (Teo, Balasubramaniam & Civil 2022). Lang (2024) found that patients with musculoskeletal injuries with limited functional mobility tend to have poor motivation to exercise and thus become non-adherent towards their HEP.

While lack of family support resulted in poor exercise adherence for some participants, receiving too much support or physical assistance from family members resulted in poor motivation and exercise participation for others. As a result, an excess amount of physical assistance from family members during exercises resulted in lowered self-confidence and, thus, a feeling of being burdensome. A study that investigated the relationship between family support and exercise adherence showed that patients were more adherent to their HEP when family members gave them less physical assistance during their exercises but were instead encouraged from afar with words of affirmation (Putri, Wati & Rekawati 2018).

One of the most significant contributors to exercise adherence is the environment patients return to after discharge from the hospital. A home environment following injury or disease can facilitate or inhibit exercise adherence. Most of these factors are difficult to change, manipulate or even remove at a given time. For some participants in our study, the chair and bed at home inhibited them from performing their prescribed home exercises, as it was too low or uncomfortable to exercise on. As a result, they considered their home environment to be a barrier to exercise adherence. Home-based exercise programmes present a unique challenge compared to centre-based programmes (Roos, Myezwa & Van Aswegen 2015). Environmental factors were the most significant barrier to exercise adherence among older adults with chronic diseases who were prescribed home-based exercise programmes (Collado-Mateo et al. 2021). Environmental factors such as space, comfort and cleanliness were considered essential when prescribing a HEP for patients diagnosed with Parkinson’s disease (Schootemeijer et al. 2020). An unconducive home environment contributed to poor adherence, as did unpredictable weather changes for specific participants in our study.

Weather changes may impact one’s ability to engage in their HEP fully. Unpredictable weather conditions may result in bodily changes, which, too, can influence one’s ability to exercise. Participants of our study reported that weather conditions that were too low or too high became barriers to exercise adherence. Low temperatures resulted in painful knee joints and the inability to engage in their exercise routine fully. A study that investigated adherence to physical activity found that altered weather conditions (too hot or too cold temperatures) could influence patients not to participate in physical activity as prescribed by their physiotherapists (Collado-Mateo et al. 2021). Furthermore, a systematic review showed that older adults prefer favourable weather conditions to become active, and the impact of weather should not be ignored when prescribing home exercises (Martín-Moya et al. 2020).

Some participants expressed time as a barrier to exercise adherence. Reference was made to work schedules and insufficient time at home, which resulted in low exercise adherence. Some participants relied on public transport to work, while others used private transport. Some participants highlighted family responsibilities as barriers to exercise adherence. A study measuring walking distance through a pedometer found that sedentary jobs prevented participants from accumulating adequate steps and decreased their exercise adherence (Holm et al. 2023). Bailey et al. (2020) report that work constraints, family responsibility and social circumstances must be considered when prescribing home exercises for patients with musculoskeletal pain.

Two facilitators of HEP adherence were reported in our study. These facilitators have adequate family support and possess a high level of motivation to exercise. Factors reported to contribute to a high level of motivation were the presence of self-discipline, the desire to return to a pre-injury state and the possibility of returning to work. Patients with positive reinforcement, high self-efficacy, self-discipline, emotional care and a strong family support system are more likely to adhere to home-based exercises (Mahmood et al. 2023). The quality of life of patients with polytrauma is primarily impacted by their ability to return to premorbid function as well as return to work (Silverstein, Higgins & Henderson 2021). According to Miller et al. (2017), social support from family members, colleagues and friends results in higher intervention adherence. Family and friends may help reduce a disease’s negative impact and encourage the patient to follow a prescribed treatment recommendation (Zhang et al. 2022). Furthermore, patients’ self-management behaviours are strongly influenced by the people with whom they live and their collective (Collado-Mateo et al. 2021).

### Study limitations

Telephonic interviews were conducted for our study, and participants were not always readily available to engage in the telephonic interviews because of personal commitments. Connectivity issues, background noises and interruptions during the phone calls were experienced during the interviews. Some participants changed their telephone numbers and thus were unreachable. Furthermore, participants diagnosed with polytrauma were limited, which increased the recruitment period.

### Recommendations

Our study explored the perspectives of patients with polytrauma injuries regarding adherence to a HEP. Research in the public sector may improve the number of participants from different socio-economic backgrounds. Exploring the perspectives of HEP adherence with other MDT members involved in the rehabilitation of polytrauma will provide a holistic viewpoint on the barriers and facilitators to polytrauma rehabilitation while providing a different lens on various strategies to overcome the barriers experienced. The development of courses on polytrauma rehabilitation will further facilitate improved clinical reasoning and understanding of polytrauma to enable a patient-centred and holistic treatment intervention. The findings of our study furthermore encourage physiotherapists to contact polytrauma-diagnosed patients who have been prescribed HEPs, within the first 3 months post-discharge from rehabilitation, to ensure adherence and to manage possible barriers that may exist.

## Conclusion

The perspectives of patients with polytrauma regarding adherence to their HEPs revealed that some barriers and facilitators influenced their exercise adherence. More barriers than facilitators were identified in our study, which limited participants’ engagement in the HEP. Barriers involve factors that can be modified or eliminated to improve adherence to exercise programmes. Physiotherapists should collaborate with their patients to ensure that prescribed HEPs are inclusive and consider patients’ demographic status, social and psychological well-being and physical and environmental profiles.
